# Fungicidal Activity of the Nitroimidazole–Thiosemicarbazide Derivative 1-[(1-methyl-4-nitroimidazol-2-yl)carbonyl]-4-(3-methylophenyl)thiosemicarbazide Against *Trichophyton* spp. Dermatophytes

**DOI:** 10.3390/pathogens15070688

**Published:** 2026-06-30

**Authors:** Sylwia Andrzejczuk, Monika Wujec, Łukasz Świątek, Urszula Kosikowska

**Affiliations:** 1 Department of Pharmaceutical Microbiology, Medical University of Lublin, Chodzki Str. 1, 20-093 Lublin, Poland; urszula.kosikowska@umlub.edu.pl; 2 Department of Organic Chemistry, Faculty of Pharmacy, Medical University of Lublin, Chodzki Str. 4A, 20-093 Lublin, Poland; 3 Department of Virology with Viral Diagnostics Laboratory, Medical University of Lublin, Chodzki Str. 1, 20-093 Lublin, Poland; lukasz.swiatek@umlub.edu.pl

**Keywords:** dermatophytes, *Trichophyton* spp., thiosemicarbazide derivatives, fungicidal effect, nitroimidazole, onychomycosis, novel antimicrobials

## Abstract

Dermatophytosis caused by *Trichophyton* spp. represents a growing public health concern, exacerbated by the increasing prevalence of difficult-to-treat infections and the emergence of resistance to standard antifungal agents, such as terbinafine. In this study, we evaluated the in vitro antifungal activity of a thiosemicarbazide derivative, 1-[(1-methyl-4-nitroimidazol-2-yl)carbonyl]-4-(3-methylphenyl)thiosemicarbazide, against a panel of anthropophilic and zoophilic *Trichophyton* spp. dermatophytes. Susceptibility testing was performed via the broth microdilution method to determine the minimum inhibitory concentrations (MICs) and minimum fungicidal concentrations (MFCs). The compound demonstrated antifungal efficacy against all the strains tested, with MIC values ranging from 31.25 to 125 µg/mL. Crucially, low MFC/MIC ratios (≤4) confirmed a fungicidal effect, which was further corroborated by microscopic analysis revealing severe hyphal damage in treated dermatophytes. The derivative exhibited consistent activity against both *T. rubrum* and *T. mentagrophytes*, including clinical isolates. To our knowledge, this is one of the first detailed evaluations of a nitroimidazole–thiosemicarbazide hybrid against *Trichophyton* spp. that combines quantitative MIC/MFC testing with morphological assessment. The tested thiosemicarbazide derivative noticeably decreased the viability of VERO and A375 cells, but even at the highest tested concentration, after 72 h of incubation, the cellular viability remained at 65%. These findings suggest that the investigated compound is a promising candidate for the development of new fungicidal drugs or disinfectants for the effective prophylaxis or management of superficial mycoses in human and veterinary medicine.

## 1. Introduction

Dermatophytes are filamentous, keratinophilic, and keratinolytic fungi that cause dermatophytoses, that is, fungal infections of the skin, scalp, and nails [1,2,3,4,5]. These fungi may be of human origin (anthropophilic, e.g., *T. rubrum*) or animal origin (zoophilic, e.g., *T. mentagrophytes*). Superficial infections of glabrous skin typically present as localized inflammation caused by opportunistic fungi such as dermatophytes. In tinea corporis, the lesions are usually round or oval, pink, single or multiple, clearly demarcated from the surrounding healthy skin, and sometimes show a paler central area. As the lesions enlarge, they may merge into extensive, irregular foci. These are often accompanied by pruritus [1,2,3,4,6,7,8].

The clinical manifestations of onychomycosis include changes in nail color and structure, increased brittleness and crumbling, subungual hyperkeratosis, and onycholysis, that is, separation of the nail plate from the nail bed. These changes largely result from the ability of dermatophytes to degrade keratin, the main structural protein of these tissues, through keratinolytic enzymes [2,3,5,8]. Most fungal infections of the skin, mucous membranes, and nails are caused by dermatophytes of the genus *Trichophyton*, mainly *T. rubrum* and *T. mentagrophytes*, and less frequently *T. interdigitale* and *T. tonsurans*. Individuals at particular risk include older adults, patients with congenital or iatrogenic immune dysfunction, those receiving long-term antibiotic therapy or other drugs that impair host defenses, and patients with chronic comorbidities (such as diabetes or thyroid disease). Additional predisposing factors include impaired peripheral circulation, occupational exposure (participation in sports, use of swimming pools, residence in dormitories or barracks, and other crowded facilities and environments), and chronic pressure and maceration associated with nonbreathable footwear [1,3,4,5,7].

Dermatophytes are currently the most common fungal pathogens worldwide. They spread efficiently through direct contact with infected individuals or animals or indirectly via fomites such as towels and clothing, posing a major public health challenge [4,5,8,9]. In Europe and Asia, *T. rubrum* infections have increased [6,10] in recent years, driven by lifestyle changes, population aging, and an increasing prevalence of onychomycosis and tinea pedis. In recent decades, shifts in the etiology of dermatomycosis, *T. tonsurans*, *T. interdigitale*/*T. mentagrophyte* complex [11,12,13,14] and the newly identified terbinafine-resistant *T. indotineae*, which often plays greater roles [11,12,13,14,15,16]. These species have fuelled outbreaks worldwide.

In southwestern Poland, adults account for >70% of dermatophytosis cases, with *T. tonsurans* causing >17% of cases [17]; in contrast, a 2019–2024 study of >1200 pediatric patients in northern Poland identified the *T. rubrum* complex as predominant [18]. Tinea capitis and tinea corporis were more prevalent among younger children, whereas tinea pedis and tinea unguium were more prevalent among adolescents. Local data from the University Clinic in Gdańsk (2011–2016) revealed that dermatophytes are responsible for >12,000 infections (7324 patients from Gdańsk, Poland, and 4729 from Grodno, Belarus), with *T. rubrum* (55% of cases) followed by the *T. mentagrophytes*/*interdigitale* complex [19]. These findings align with global trends, including a Chinese study of >30,000 clinical cultures (2018–2023), in which *T. rubrum* reached an approximately 90% prevalence [20]. Dermatophytosis affects 20–25% of the population worldwide [6,11,12,13,14,16,21,22]. *T. indotineae,* closely related to *T. mentagrophytes* and *T. interdigitale*, is spreading rapidly beyond South Asia [12,22], accounting for nearly 40% of UK (>157 cases, 2017–2024) reference laboratory dermatophyte isolates [11] and exhibiting terbinafine resistance [11,12,14,15,16,21].

Given these trends, developing new, more effective prevention and treatment strategies, alongside innovative antifungal therapies, has become an urgent priority for contemporary pharmacy and medicine. Despite advances in topical and systemic antifungals (particularly those active against dermatophytes), the global emergence and spread of terbinafine-resistant *T. indotineae* and other *Trichophyton* spp. underscore the critical need for novel fungicides with alternative mechanisms of action [14,23]. Hybrid molecules combining nitroimidazole and thiosemicarbazide moieties have recently been recognized as promising platforms for broad-spectrum antifungal drug candidates [24,25].

Despite the wide availability of topical and systemic antifungal agents, treating dermatophytoses remains challenging. Therapeutic failure, relapses, prolonged treatment durations, and adverse effects are still common, with increasing concerns about emerging resistance, particularly to terbinafine, in *Trichophyton* spp., including *T. indotineae* and certain *T. rubrum* isolates [4,6,7,10,12,20,21,26,27,28]. These limitations highlight the need for novel fungicidal compounds with reliable activity against dermatophytes and potential applications in both human and veterinary medicine.

Thiosemicarbazide derivatives and nitroimidazole-based compounds have garnered attention for their broad antimicrobial effects, including those against pathogenic fungi. Hybrid molecules that combine these pharmacophores into a single scaffold may offer enhanced antifungal potency and selectivity toward dermatophytes [26,27]. These agents could serve not only to treat and prevent superficial mycoses but also as active ingredients in disinfectants and antiseptic formulations to reduce the number of environmental reservoirs of dermatophytes.

Therefore, this study evaluated the antifungal and fungicidal activities of 1-[(1-methyl-4-nitroimidazol-2-yl)carbonyl]-4-(3-methylphenyl)thiosemicarbazide against *Trichophyton* spp. dermatophytes. We assessed its in vitro activity profile and potential as a lead candidate for novel antifungal formulations in human and veterinary applications.

## 2. Materials and Methods

### 2.1. Thiosemicarbazide Derivative

**Figure 1:**
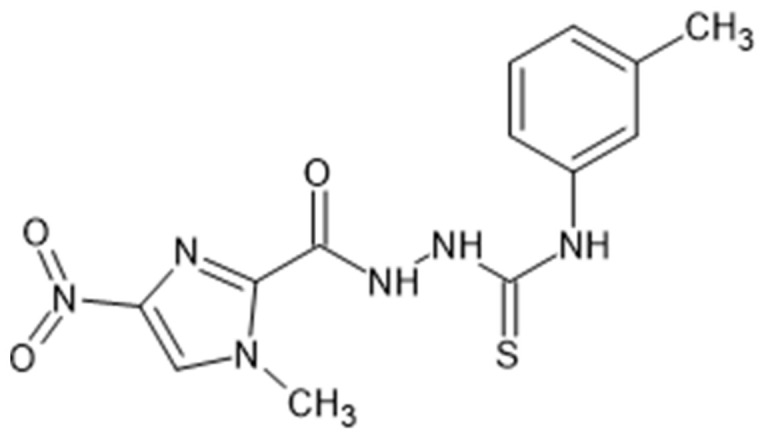
Chemical structure of the 1-[(1-methyl-4-nitroimidazole-2-yl)carbonyl]-4-(3-methylphenyl)thiosemicarbazide derivative investigated against *Trichophyton* spp. dermatophytes.

The thiosemicarbazide derivative was synthesized by reacting 1-methyl-4-nitroimidazole-2-carboxylic acid hydrazide with 3-methylphenyl isothiocyanate in anhydrous ethanol at a temperature just below the boiling point for 1 h (Figure 1). The precipitate was filtered after cooling, washed with hot distilled water and diethyl ether, air-dried, and recrystallized from 96% ethanol. Full synthetic details are available in prior publications [25,29,30], where this compound was designated no. 16 [31].

### 2.2. Antifungal Activity Assay

The antifungal activity of 1-[(1-methyl-4-nitroimidazole-2-yl)carbonyl]-4-(3-methylphenyl)thiosemicarbazide was evaluated via the broth microdilution method in accordance with the European Committee on Antimicrobial Susceptibility Testing (EUCAST) guidelines [31,32,33,34]. Fungal cultures were grown aerobically on solid Sabouraud agar (Biomaxima, Lublin, Poland) at 25 ± 2 °C for 5–10 days prior to testing. Reference strains from the American Type Culture Collection (ATCC) included *T. interdigitale* ATCC 9533 (formerly *T. mentagrophytes*) and *T. rubrum* ATCC 28188, along with clinical isolates (*T. mentagrophytes* complex, *T. rubrum*, and *T. tonsurans*) from diverse body sites (e.g., toenails, facial/neck/groin/hand/foot skin) found in the museum collection of the Department of Pharmaceutical Microbiology at the Medical University of Lublin, Poland.

A stock solution of the tested compound (50 mg/mL) was prepared in 1 mL of dimethyl sulfoxide (DMSO, POCH, Gliwice, Poland). A working solution (2000 µg/mL) was then prepared in sterile Mueller–Hinton broth (MHB, Biomaxima, Lublin, Poland) enriched with 2% glucose (Novo Nordisk, Warsaw, Poland). This solution was used to test the sensitivity of fungi to antifungal substances. To determine antifungal activity via the minimum inhibitory concentration (MIC), 96-well microtiter plates (Medlab, Raszyn, Poland) were used. Briefly, 200 µL of the 2000 µg/mL solution was added to the first row; 100 µL of sterile MHB + 2% glucose was added to subsequent rows. Serial twofold dilutions were prepared by transferring 100 µL from each row of wells to the next row (with thorough mixing), yielding final concentrations ranging from 3.91 to 2000 µg/mL. Fluconazole (Glentham Life Sciences, Corsham, UK) was tested at 0.049–100 µg/mL. All tests were performed in triplicate across three independent experiments, with interexperimental variability <2-fold dilution, per EUCAST guidelines. Representative data are shown.

Suspensions of the tested dermatophytes were prepared in an aseptic environment in accordance with the EUCAST E. Def 9.4 standard [32,35]. Sterile equipment and reagents were used for this purpose. A small amount of material was collected from a fresh fungal culture via a moistened swab dipped in 0.85% NaCl solution and transferred to tubes containing 5 mL of distilled water supplemented with 0.1% Tween-20 (Chemland, Stargard, Poland). After thorough mixing, the suspension was vortexed for 15 s and then transferred to a sterile syringe fitted with an 11 µm pore-diameter filter (Bionovo, Legnica, Poland) for filtration. The filtrate was collected in a sterile test tube to remove hyphal fragments. The suspension was adjusted to a density of 0.5 on the McFarland scale (2–5 × 10^6^ CFU/mL) in distilled water. The suspensions of the tested fungi obtained in this way were then diluted in MHB + 2% glucose medium to a final density of 2–5 × 10^5^ CFU/mL. This mixture was then introduced into all the wells of the plate in a 100 µL volume via an automatic pipette. While determining the activity of the compound against the tested *Trichophyton* spp. strains, the following steps were carried out: (a) checking the viability of each of the tested fungal strains by adding 100 µL of the suspension to three wells containing pure MHB + 2% glucose medium without the tested compound; (b) checking the purity of the MHB + 2% glucose medium by placing 100 µL of sterile medium without the tested compound or fungi in the wells of a titration plate; and (c) checking the tested compound at the same series of dilutions in MHB + 2% glucose medium without adding the fungal suspension [33].

The microplates were prepared and incubated in a humid chamber at 25 ± 2 °C under aerobic conditions for 3–4 days. After this time, the growth of the dermatophytes in the wells was analyzed via a Bio-Tek ELx800 spectrophotometer (Biokom, Janki, Poland) at 490 nm and via macroscopic observation of turbidity. The endpoints were defined as the lowest concentration of the compound that completely inhibited growth, compared with growth in control wells containing only the quality control agent and no test compounds. All evaluations were performed in triplicate.

The measurements were then processed via the Gen5 version. 3.03.14 (Biokom, Janki, Poland). Fungal growth was indicated by visible turbidity in the microplate wells. The effects of 1-[(1-methyl-4-nitroimidazol-2-yl)carbonyl]-4-(3-methylphenyl)thiosemicarbazide against *Trichophyton* spp. dermatophytes were assessed on the basis of the minimum inhibitory concentration (MIC) and minimum fungicidal concentration (MFC). The MIC was defined as the lowest concentration of the test compound in wells that inhibited visible fungal growth by ≥90%. The lowest concentration of the test compound was 50% (MIC_50_—for endpoints determined spectrophotometrically with a 50% growth inhibition endpoint) or 90% (MIC_90_—for endpoints determined spectrophotometrically with a 90% growth inhibition endpoint) [36]. A reduction in the growth of the test fungi was observed after 5–7 days of incubation compared with the growth of the control fungi at 25 ± 2 °C under aerobic conditions. The MFC was defined as the lowest compound concentration that did not change or exhibited approximately 99.0–99.5% killing activity, as confirmed by microscopic observation and subculture on drug-free media [37]. Control wells containing fungi but no test compound were used to verify normal fungal growth. On the basis of the obtained results and the MFC/MIC ratio, the fungicidal (MFC/MIC ≤ 4) or fungistatic (MFC/MIC > 4) activity of the tested compound against dermatophytes was determined [32,35].

### 2.3. Cytotoxicity Screening

Cytotoxicity was tested in the BSL-2 laboratory following previously described experimental procedures [38]. The noncancerous VERO (ATCC, CCL-81) and cancer-derived A375 (ATCC, CRL-1619) and MDA-MB-231 (ATCC, HTB-26) cells were used in this study. The media used for in vitro culture included Dulbecco’s modified Eagle’s medium (DMEM; Corning, Tewksbury, MA, USA) for VERO and A375 cells and modified Eagle’s medium (MEM; Corning) for MDA-MB-231 cells. The media were supplemented with antibiotics (penicillin–streptomycin solution, Corning) and fetal bovine serum (FBS, Corning) at 10% (passaging) and 2% (maintenance/experiments). Phosphate-buffered saline (PBS) and trypsin were obtained from Corning, while 3-(4,5-dimethylthiazol-2-yl)-2,5-diphenyltetrazolium bromide (MTT) was purchased from Sigma-Aldrich (Merck KGaA, Darmstadt, Germany). The mixture was incubated at 37 °C in 5% CO_2_ (Panasonic Healthcare Co., Tokyo, Japan). The cells were subcultured in T25 flasks (EasYFlasks, Nunc, Thermo Fisher Scientific, Waltham, MA, USA) and then passaged into 96-well plates (Falcon, Corning) to produce cell monolayers. The number of passages for all the cell lines used in this research did not exceed 20. The thiosemicarbazide derivative was dissolved (50 mg/mL) in DMSO (cell culture grade, PanReac Applichem, Darmstadt, Germany) to produce stock solutions, which were stored frozen (−23 °C) until analysis. Cytotoxicity was assessed via a microculture tetrazolium (MTT) assay. Briefly, monolayers of appropriate cells in 96-well plates were incubated for 72 h with serial dilutions of the thiosemicarbazide derivative in culture media. Afterward, the media were removed, the cell monolayers were washed with PBS, and MTT-supplemented medium was added. Following a 3 h incubation, the formazan product was dissolved, and the solution was incubated overnight. Moreover, the toxicity of the use of DMSO as a solvent for stock solutions was evaluated. The absorbance was measured (at 540 and 620 nm) via a Synergy H1 Multi-Mode Microplate Reader (BioTek Instruments, Inc., Winooski, VT, USA) with Gen5 software (ver. 3.09.07; BioTek Instruments, Inc., Winooski, VT, USA), and the results were exported to GraphPad Prism (version 10.2.0, San Diego, CA, USA) for analysis. The experiments were performed in triplicate across 3 independent experiments. The statistical significance of differences between means (mean cellular viability) was determined via two-way ANOVA followed by Tukey’s post hoc tests. A *p* value of <0.05 was considered to indicate statistical significance.

## 3. Results

### 3.1. Antifungal Activity Against Trichophyton spp. Dermatophytes

The antifungal activity of 1-(1-methyl-4-nitroimidazol-2-yl)carbonyl-4-(3-methylphenyl)thiosemicarbazide was evaluated against a panel of *Trichophyton* spp. strains, including both anthropophilic (*T. rubrum*, *T. tonsurans*) and zoophilic (*T. mentagrophytes* complex) species.

The obtained MIC values ranged from 31.25 to 125 µg/mL (Table 1), indicating antifungal inhibitory activity. Notably, the compound was highly effective against clinical isolates of *T. rubrum*, the most common etiological agent of onychomycosis, with MICs consistently lower than 125 µg/mL.

To determine the nature of the antifungal effect, the MFCs were assessed. On the basis of the MIC_50_ values (ranging from 31.25 to 250 µg/mL), the MIC_90_ values (ranging from 62.5 to 250 µg/mL), and the calculated MFC/MIC ratios (MFC/MIC = 1), the compound was fungicidal rather than fungistatic toward the majority of the dermatophyte strains tested. The most favorable activity was observed against the *T. rubrum* ATCC 28188 reference strain (MIC_50_ = 31.25 µg/mL, MIC_90_ = 62.5 µg/mL, MFC/MIC = 2). Overall, the compound demonstrated consistent fungicidal properties (MFC/MIC ≤ 4) against both reference and clinical isolates, including *T. rubrum* strains, which are the leading epidemiological cause of onychomycosis worldwide. This activity was confirmed microscopically by an inhibitory effect on fungal growth and an absence of hyphae and/or spores produced by the tested reference strains and clinical isolates.

### 3.2. Microscopic Analysis of Trichophyton spp. Morphology Under the Influence of the Compound Tested

**Figure A1:**
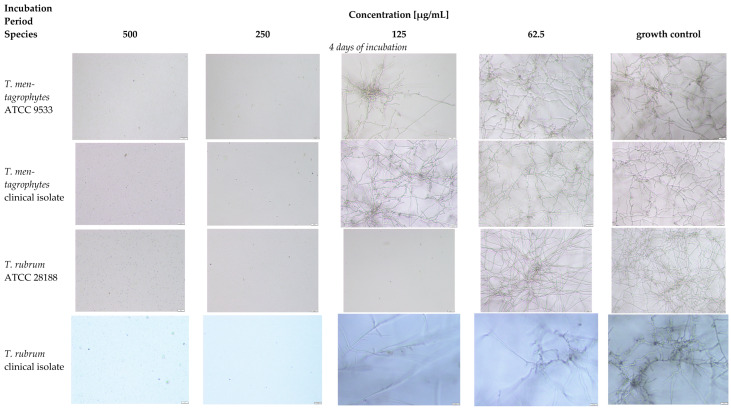

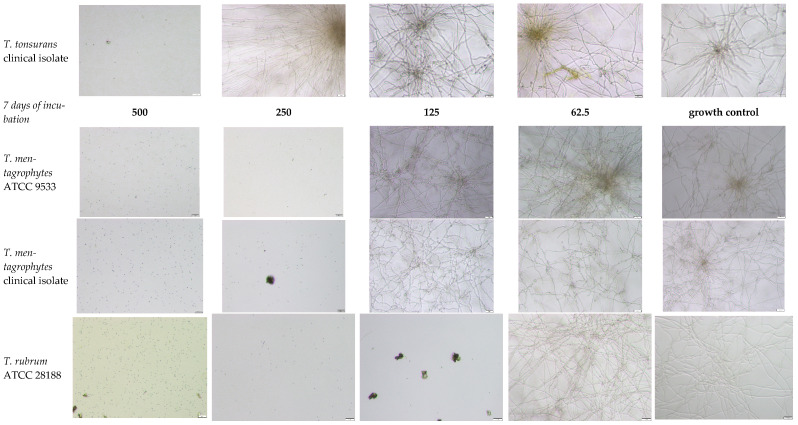

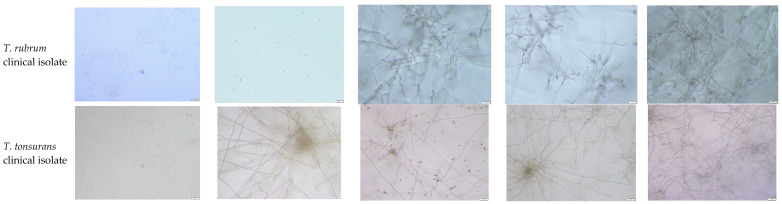
Microscopic observation of the thiosemicarbazide derivative against *Trichophyton* spp. dermatophytes investigated (4 and 7 days at 25 ± 2 °C under aerobic conditions). Abbreviations: 2000× magnification, 20 µm scale bar, Olympus DP22 automated inverted light microscope, CellSens Dimensions 2.3 software, Olympus Corporation; growth control refers to the growth of fungi without the presence of the tested thiosemicarbazide derivative.

To further elucidate the antifungal effects of 1-(1-methyl-4-nitroimidazol-2-yl)carbonyl-4-(3-methylphenyl)thiosemicarbazide, microscopic observations of *Trichophyton* spp. hyphal growth were performed following exposure to the compound. Following the MIC readings, the incubation process continued for up to 7 days in a humid chamber under aerobic conditions at 25 ± 2 °C. After 4 and 7 days of incubation, a microscopic evaluation of the impact of the test compound on the growth and formation of hyphae and spores of dermatophytes in the genus *Trichophyton* (Figure A1) was performed. Following incubation, the presence of hyphae and spores in individual wells of a multiwell plate was examined under 2000× magnification via an Olympus DP22 inverted automatic light microscope equipped with CellSens Dimensions 2.3 software (Olympus Corporation, Tokio, Japan). Examples of the microscopic evaluation of the effects of selected concentrations of 1-[(1-methyl-4-nitroimidazol-2-yl)carbonyl]-4-(3-methylphenyl)thiosemicarbazide on the presence of hyphae and spores of dermatophytes are presented in Figure A1.

The control samples cultivated in the tested compound- or fluconazole-free media retained the typical morphology of dermatophytes, with long, regular, branched hyphae and smooth cell walls. In contrast, fungal cells exposed to the tested thiosemicarbazide derivative presented pronounced structural alterations, including irregular branching, cytoplasmic granulation, and marked vacuolization, which was particularly evident in the *T. rubrum* ATCC 28188 strain. At concentrations equal to or exceeding the MIC of the tested derivative, only single, nongerminating conidia or no conidia at all were observed in the microscopic field, and hyphae were also absent.

### 3.3. Influence on the Cell Lines

**Figure 2:**
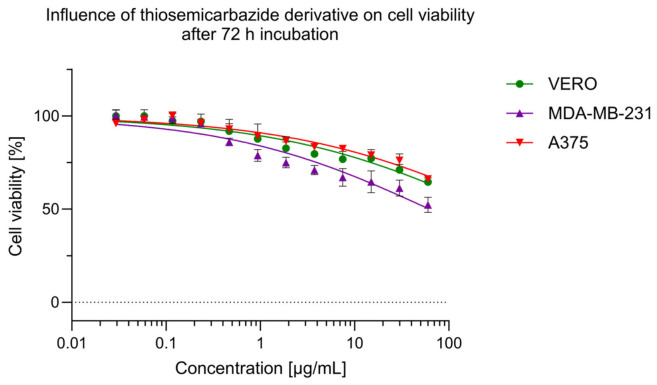
Dose-response influence of the 1-[(1-methyl-4-nitroimidazole-2-yl)carbonyl]-4-(3-methylphenyl)thiosemicarbazide derivative on VERO, A375 and MDA-MB-231 cell lines.

When 1-[(1-methyl-4-nitroimidazol-2-yl)carbonyl]-4-(3-methylphenyl)thiosemicarbazide was tested at 125 µg/mL, a small amount of crystals were observed in the culture medium. It was not possible to increase the solubility of the thiosemicarbazide derivative by increasing the DMSO concentration because of potential toxic effects on cell lines. Thus, cytotoxicity was assessed at concentrations up to 62.5 µg/mL. The thiosemicarbazide derivative showed a dose-dependent cytotoxic effect on the tested cell lines, as shown in Figure 2, with VERO and A375 cells exhibiting a similar pattern of cytotoxicity, decreasing the mean cellular viability to 65% (±2 pp) at 62.5 µg/mL. Compared with VERO and A375 cells, MDA-MB-231 cells were more sensitive to the test compounds, with a mean cellular viability ranging from 0.98 to 62.5 µg/mL, which was significantly lower (*p* < 0.05). At the highest tested concentration, the mean viability of MDA-MB-231 cells was 52% (±4 pp).

## 4. Discussion

When the literature on this topic is analyzed, several studies have described nitrogen-containing heterocyclic compounds with antifungal activity [25,31,39,40,41]. This is not surprising, given that many clinically used drugs contain nitrogen heterocycles. Güzeldemirci et al. [39] reported the synthesis of new or modified antifungal and antibacterial agents on the basis of a core structure comprising two heterocyclic rings, imidazole and thiazole. Among these derivatives, only a single compound exhibited high activity against *T. rubrum* and *Microsporum audouini*, comparable to that of ketoconazole. The same group subsequently developed new thiosemicarbazide derivatives, as well as cyclic analogs of 1,2,4-triazole and 1,3,4-thiadiazole containing imidazothiazole moieties [40]. All of these compounds were subjected to biological evaluation, and two thiosemicarbazides displayed antifungal activity against yeast-like fungi (*Candida parapsilosis* ATCC 22019 and *Candida krusei* ATCC 6258) and dermatophytes of the genus *Trichophyton*, including *T. erinacei* NCPF 375 and *T. tonsurans* NCPF 245, with MIC values of 64 μg/mL. In another study, Terzioğlu Klip et al. [31] obtained S-(4,5-disubstituted-1,2,4-triazolo)acetic acid hydrazide and converted it into the corresponding thiosemicarbazide derivatives by reacting it with aliphatic and aromatic isothiocyanates. The resulting compounds were tested for antifungal activity against *Microsporum gypseum* (NCPF 580), *Microsporum canis*, *T. mentagrophytes*, and *Candida albicans* (ATCC 10231). Two derivatives, one bearing a 4-bromophenyl substituent and the other bearing a 4-nitrophenyl group at the 4-position of the thiosemicarbazide moiety, exhibited broad-spectrum antifungal activity, with MIC values of 4–8 μg/mL. In this case, the antifungal effect resulted from the combined influence of the triazole/thiosemicarbazide framework and strongly electron-withdrawing *para* substituents. In contrast, the compound investigated in the present study contains a 1-methyl-4-nitroimidazole pharmacophore linked to a 3-methylphenyl-substituted thiosemicarbazide moiety. The presence of the 3-methyl group may influence antifungal activity through several mechanisms. As a weak electron-donating substituent, the methyl group increases the electron density of the aromatic ring and may influence the acidity of adjacent NH groups within the thiosemicarbazide fragment. Furthermore, the *meta-methyl* substituent increases lipophilicity and steric bulk, which may enhance penetration through the lipid-rich fungal cell envelope and affect interactions with biological targets. Unlike *para*-substituted electron-withdrawing groups such as nitro or bromo groups, the *meta-methyl* group does not exhibit strong resonance effects, suggesting that the observed activity of the present compound is more likely attributable to a favorable balance among lipophilicity, steric effects, and the contribution of the nitroimidazole moiety.

It is therefore plausible that the antifungal activity observed in this study arises from the synergistic combination of the nitroimidazole pharmacophore and the thiosemicarbazide scaffold rather than from the isolated effect of the 3-methyl substituent alone. Further structure–activity relationship studies involving direct comparisons of meta-methyl, para-bromo, and para-nitro analogs will be needed to clarify the precise role of electronic and steric effects in determining antifungal potency. Yamaguchi et al. [41] further demonstrated that introducing a thiosemicarbazide system into a molecule can increase its antifungal activity by up to 15-fold. Taken together, these findings indicate that the incorporation of a thiosemicarbazide scaffold and appropriately substituted aromatic rings is a promising strategy for enhancing antifungal potency against dermatophytes and yeast-like fungi.

Although no dedicated mechanistic assay was performed in the present work, the observed fungicidal activity and morphological alterations suggest that the tested nitroimidazole–thiosemicarbazide derivative affects fungal cellular integrity. However, the precise molecular mechanism remains unknown and needs further research [42,43]. Previous studies on structurally related thiosemicarbazide/thiosemicarbazone and nitroimidazole derivatives proposed mechanisms involving membrane perturbation, oxidative stress induction, and redox imbalance, although these effects have not yet been verified for the present compound [24,41,44]. Such effects have been reported for related antimicrobial scaffolds, although they were not tested here [45]. Yamaguchi et al. [41] demonstrated that a camphene–thiosemicarbazide derivative active against *T. mentagrophytes* induced morphological alterations and impaired Calcofluor White staining, suggesting effects on chitin-containing structures, whereas ergosterol did not appear to be the primary target. Thiosemicarbazide-based scaffolds are increasingly recognized as privileged structures in the design of antifungal drugs, with several series demonstrating low-to-moderate minimum inhibitory concentrations (MICs) against dermatophytes and yeast-like fungi [24]. In this context, the activity profile of our compound, including its fungicidal effect on *Trichophyton* spp. dermatophytes, is at least comparable to and, in some cases, more favorable than the MIC values of 31.25–500 µg/mL reported for previously described thiosemicarbazide derivatives [25].

A comparison of our results with literature data provides valuable insights into the structure-activity relationships within this class of compounds. While previous studies on thiosemicarbazide derivatives reported MIC values against dermatophytes ranging from 125 to 2000 µg/mL for *T. mentagrophytes* and from 31.25 to 2000 µg/mL for *T. rubrum* [25], the 1-[(1-methyl-4-nitroimidazol-2-yl)carbonyl]-4-(3-methylphenyl)thiosemicarbazide investigated in this study demonstrated good potency, with MIC values in the range of 31.25–125 µg/mL against both species (with clearly lower MIC values against *T. rubrum*). This enhanced activity may be attributed to the specific combination of the 1-methyl-4-nitroimidazole moiety and the 3-methylphenyl substituent. Unlike the 4-bromophenyl and 4-nitrophenyl derivatives described by Terzioğlu Klip et al. [31], which contain strong electron-withdrawing groups, the presence of a methyl group in the meta position of the phenyl ring appears to favorably influence the interaction of the molecule with fungal targets. Furthermore, the nitroimidazole core itself is known to contribute to antimicrobial efficacy, potentially through mechanisms involving nitro-group reduction and the subsequent generation of toxic intermediates, a pathway well established for nitroimidazole drugs but less explored in the context of dermatophyte inhibition.

A critical finding of this study is the fungicidal nature of the tested compound against *Trichophyton* spp., as evidenced by the low MFC/MIC ratios. In clinical practice, fungicidal agents are generally preferred over fungistatic agents for the treatment of dermatophytoses, particularly in immunocompromised patients or in cases of onychomycosis where eradication of the pathogen from the keratinized matrix is difficult [2,3,7,22,27,46,47]. While many azoles primarily exert fungistatic effects, our ability to kill dermatophyte cells at concentrations near the inhibitory level suggests the potential for higher cure rates and a lower risk of relapse [1,7,27]. This profile is particularly promising in the context of the increasing prevalence of difficult-to-treat infections caused by *T. indotineae* and terbinafine-resistant *T. rubrum* strains [10,11,12,13,14,15,21,47,48]. The clinical importance of a fungicidal profile is further emphasized by the rapid worldwide emergence of *T. rubrum* strains resistant to terbinafine, which are often associated with squalene epoxidase mutations and elevated MICs. This results in recalcitrant cases of tinea corporis and cruris [14,23,49]. Novel agents that target alternative pathways and retain activity against *Trichophyton* spp., regardless of the mechanism of terbinafine resistance, could be valuable options for the management of difficult-to-treat dermatophytoses and for antifungal stewardship [14,23].

A noncancerous VERO cell line was selected to study the cytotoxicity of the derivative 1-[(1-methyl-4-nitroimidazol-2-yl)carbonyl]-4-(3-methylphenyl)thiosemicarbazide. VERO cells were selected because they can be maintained and passaged without signs of senescence, provide reliable, repeatable results, and are commonly used in cytotoxicity studies and in vitro efficacy testing [50,51,52]. Moreover, VERO cells are endorsed by ISO experts as suitable for in vitro cytotoxicity testing [53]. For cytotoxicity comparisons, two cancer cell lines—MDA-MB-231 and A375—were selected, originating from breast adenocarcinoma and malignant melanoma, respectively. The evaluation of the impact of the 1-[(1-methyl-4-nitroimidazol-2-yl)carbonyl]-4-(3-methylphenyl)thiosemicarbazide derivative on the tested cell lines revealed a potential cytotoxic influence, reducing the viability of the MDA-MB-231 or 65% VERO, and A375 cells to 52% at the highest tested concentration. When fluconazole was tested in VERO cells, cytotoxicity after 24 h of incubation was observed at 1306 μM fluconazole (approx. 400 µg/mL), reducing viability to 86%. At 2612.1 μM, viability was reduced to 35%. This effect was likely due to fluconazole-induced cell necrosis, increased reactive oxygen species (ROS), and DNA damage [43]. Fluconazole showed greater cytotoxicity against L929 cells (murine fibroblasts), with reduced cell viability observed at 24 and 48 h of incubation starting at 62.5 µg/mL, and at 250 µg/mL, it was reduced by more than 90%. Interestingly, miconazole showed greater cytotoxicity at 15.6 µg/mL, reducing L929 viability to less than 60% [54] Miconazole also showed greater cytotoxicity against HaCaT cells (human keratinocytes) than terbinafine, with noticeable cytotoxicity at a concentration of 6.25 µg/mL. Both miconazole and terbinafine stimulate ROS in keratinocytes, potentially inducing oxidative stress and cell death [55]. Considering the reported cytotoxicity of approved antifungals such as fluconazole, miconazole, and terbinafine, the observed effects of the 1-[(1-methyl-4-nitroimidazol-2-yl)carbonyl]-4-(3-methylphenyl)thiosemicarbazide derivative on VERO, A375, and MDA-MB-231 do not preclude its potential antifungal applications. However, the authors acknowledge the limitations of the applied cytotoxicity methodology, the limited selection of cell lines, and the need for further testing to evaluate the safety of the new antifungal, 1-[(1-methyl-4-nitroimidazol-2-yl)carbonyl]-4-(3-methylphenyl)thiosemicarbazide, including in vitro skin irritation and other bioassays. Importantly, future studies should include a human keratinocyte cell line to better assess skin cytotoxicity associated with potential topical application.

From a practical perspective, the broad spectrum of activity and fungicidal mechanism of the investigated thiosemicarbazide derivative open several potential avenues for application. Its high efficacy against zoophilic and anthropophilic dermatophytes suggests that it could be developed not only as a therapeutic agent for superficial mycoses in humans but also for veterinary use. Furthermore, given the environmental stability of dermatophyte arthrospores, the compound could serve as an active ingredient in surface disinfectants for high-risk areas such as swimming pools, gyms, and veterinary clinics. Future studies should focus on evaluating the safety profile of this molecule in mammalian cells, assessing its skin permeation properties, and developing optimal topical formulations, such as lacquers or creams, to fully exploit its therapeutic potential.

Current topical therapies for onychomycosis, such as efinaconazole and tavaborole, exhibit variable fungicidal versus fungistatic behavior against *Trichophyton* spp., with efinaconazole demonstrating potent fungicidal activity in keratin-containing media, whereas tavaborole predominantly exhibits fungistatic properties [56,57]. Collectively, these data indicate that the ability to exhibit fungicidal activity in a keratin-rich environment is an important factor in achieving a clinical cure. These findings suggest that nitroimidazole–thiosemicarbazide derivatives with confirmed fungicidal activity could be promising candidates for topical formulations to treat nails or skin [56].

This study has several strengths, including the use of both reference and clinical *Trichophyton* isolates, determination of MIC and MFC values according to standard microdilution protocols, and a combination of quantitative susceptibility testing with microscopic assessment of hyphal damage. The observed morphological alterations should therefore be interpreted only as indicators of the cellular response to the compound and not as definitive evidence of a specific antifungal mechanism. The precise molecular mechanism and proposed effects, such as membrane perturbation or oxidative stress, should be considered in future studies. However, this work has several limitations. These include the lack of biofilm- or keratin-related models and the need for in vivo evaluation; mechanistic studies are necessary to determine the exact cellular targets involved. These issues should be addressed in future studies before any drug is developed.

## 5. Conclusions

The tested compound, 1-(1-methyl-4-nitroimidazol-2-yl)carbonyl-4-(3-methylphenyl)thiosemicarbazide, demonstrated potent antifungal activity, exhibiting lethal effects against dermatophytes of the genus *Trichophyton*. The data obtained identify this molecule as a fungicidal agent, offering a distinct advantage over purely fungistatic alternatives. Consequently, it represents a promising candidate for the development of novel pharmaceuticals to prevent and treat superficial mycoses caused by anthropophilic and zoophilic dermatophytes in both humans and animals. In addition to clinical therapy, its biological activity provides grounds for potential applications in the medical, pharmaceutical, agricultural, and veterinary industries, including its use as an effective disinfectant, antiseptic, or preservative. Future research will focus on evaluating its safety profile and in vivo efficacy to further validate its versatility and clinical utility. Owing to its consistent fungicidal activity against *Trichophyton* spp., the tested nitroimidazole–thiosemicarbazide derivative shows promise as a lead compound for the further preclinical development of topical antifungals or disinfectants targeting dermatophytoses in humans and/or animals.

## Figures and Tables

**Table 1:** Fungicidal activity of 1-[(1-methyl-4-nitroimidazol-2-yl)carbonyl]-4-(3-methylphenyl)thiosemicarbazide on the basis of MIC and MFC values against reference strains and clinical isolates of dermatophytes belonging to the genus *Trichophyton*.

Strain			MIC_50_ [µg/mL]	MIC_90_ [µg/mL]	MFC [µg/mL]	MFC/ MIC_50_	MFC/ MIC_90_	Inhibitory Effect
reference strains	*T. interdigitale* ATCC 9533		125	125	125	1	1	(+)
	*T. rubrum* ATCC 28188		31.25	62.5	62.5	2	2	(+)
clinical isolates	isolate no. 1	*T. mentagrophytes* complex	125	250	250	2	2	(+)
	isolate no. 2	*T. rubrum*	62.5	125	250	2	4	(+)
	isolate no. 3	*T. tonsurans*	250	250	250	1	1	(+)

Abbreviations: *Trichophyton interdigitale* ATCC 9533—formerly *T. mentagrophytes* ATCC 9533; MIC_50_ and MIC_90_—the lowest concentration of the test compound at which 50% (MIC_50_) or 90% (MIC_90_) fungal growth inhibition was observed; (+)—fungicidal activity of the studied compound; values of MICs_50_ or MICs_90_ and MFC for fluconazole were as follows: 50–100 µg/mL, 12.5–100 µg/mL, and 25–100 µg/mL for *T. mentagrophytes*; 12.5 µg/mL, 25–50 µg/mL, and 100 µg/mL for *T. rubrum*; and 100 µg/mL, 100 µg/mL, and 100 µg/mL for *T. tonsurans*. The experiment was performed in triplicate, and representative data are shown.

## Data Availability

The raw data supporting the conclusions of this article will be made available by the authors upon request.

## Abbreviations

The following abbreviations are used in this manuscript:

MIC	minimal inhibitory concentration
MFC	minimal fungicidal concentration
MFC_50_	the lowest concentration that establishes a predetermined reduction of 50% of fungal growth after 5–7 days of incubation, as compared to the growth in the control
MFC_90_	the lowest concentration that establishes a predetermined reduction of 90% of fungal growth after 5–7 days of incubation, as compared to the growth in the control
